# Sample-Pooling Strategy for SARS-CoV-2 Detection among Students and Staff of the University of Sannio

**DOI:** 10.3390/diagnostics11071166

**Published:** 2021-06-26

**Authors:** Immacolata Polvere, Elena Silvestri, Lina Sabatino, Antonia Giacco, Stefania Iervolino, Teresa Peluso, Rosa Guida, Lucrezia Zerillo, Romualdo Varricchio, Silvia D’Andrea, Serena Voccola, Jessica Raffaella Madera, Alberto Zullo, Romania Stilo, Pasquale Vito, Tiziana Zotti

**Affiliations:** 1 Dipartimento di Scienze e Tecnologie, Università Degli Studi del Sannio, Via dei Mulini, 82100 Benevento, Italy; immapolvere88@gmail.com (I.P.); silves@unisannio.it (E.S.); sabat@unisannio.it (L.S.); antonia.giacco@unisannio.it (A.G.); stefania.iervolino@unisannio.it (S.I.); teresa.peluso@unisannio.it (T.P.); rosa.guida@unisannio.it (R.G.); lzerillo@unisannio.it (L.Z.); jrmadera@unisannio.it (J.R.M.); albzullo@unisannio.it (A.Z.); romsti@unisannio.it (R.S.); 2 Genus Biotech, Università degli Studi del Sannio, SS Appia, 82030 Apollosa, Italy; romualdovar@outlook.it (R.V.); silvia.dandrea@tecnobios.com (S.D.); serena.voccola@tecnobios.com (S.V.); 3 Consorzio Sannio Tech, SS Appia, 82030 Apollosa, Italy

**Keywords:** SARS-CoV-2, sample pooling, RT-qPCR

## Abstract

Since the beginning of the Severe Acute Respiratory Syndrome Coronavirus 2 (SARS-CoV-2) pandemic, it has been clear that testing large groups of the population was the key to stem infection and prevent the effects of the coronavirus disease of 2019, mostly among sensitive patients. On the other hand, time and cost-sustainability of virus detection by molecular analysis such as reverse transcriptase-quantitative polymerase chain reaction (RT-qPCR) may be a major issue if testing is extended to large communities, mainly asymptomatic large communities. In this context, sample-pooling and test grouping could offer an effective solution. Here we report the screening on 1195 oral-nasopharyngeal swabs collected from students and staff of the Università degli Studi del Sannio (University of Sannio, Benevento, Campania, Italy) and analyzed by an in-house developed multiplex RT-qPCR for SARS-CoV-2 detection through a simple monodimensional sample pooling strategy. Overall, 400 distinct pools were generated and, within 24 h after swab collection, five positive samples were identified. Out of them, four were confirmed by using a commercially available kit suitable for in vitro diagnostic use (IVD). High accuracy, sensitivity and specificity were also determined by comparing our results with a reference IVD assay for all deconvoluted samples. Overall, we conducted 463 analyses instead of 1195, reducing testing resources by more than 60% without lengthening diagnosis time and without significant losses in sensitivity, suggesting that our strategy was successful in recognizing positive cases in a community of asymptomatic individuals with minor requirements of reagents and time when compared to normal testing procedures.

## 1. Introduction

The zoonotic spread of the novel beta coronavirus SARS-CoV-2 is characterized by high transmission rates, respiratory symptoms and nonspecific manifestations, similar to those due to seasonal influenza virus infection. In the most sensitive patients, SARS-CoV-2 infection can cause interstitial pneumonia, acute respiratory distress syndrome (ARDS), and multiorgan damage leading to death [1,2,3]. One year after the outbreak, there are no therapeutically effective antiviral drugs and, still, the fatality rate is higher in elderly patients over 60 years of age and/or with comorbidities [4,5]. Due to its speed of transmission, the large prevalence of asymptomatic cases, the severity of symptoms in the most fragile patients and the pressure exerted on the healthcare system, the SARS-CoV-2 pandemic has imposed, on the one hand, the need for an accurate method to detect and characterize the etiological agent in the host at a molecular level, and, on the other hand, the possibility to test as large as possible groups of population repeatedly (6). Plenty of diagnostic tools were developed during the first wave of COVID-19 outbreak, playing a crucial role in stemming the infection and thereby allowing rapid detection of newly infected individuals at the point of care, their isolation and contact tracing [6,7]. While immunological tests describe the antibody response to infection, the direct identification of the virus in respiratory or saliva specimens can be carried out by antigenic tests or by amplification of its genome through RT-qPCR or ddPCR. The latter approaches, currently called “molecular tests”, are indicated as the gold standard in defining positive cases when the viral load is low [8,9,10,11,12]. Nevertheless, individual screening of large asymptomatic cohorts by RNA extraction and RT-qPCR can be expensive and wasteful when pathogens are present in the population at low carriage rates. As recommended by the FDA (https://www.fda.gov/medical-devices/coronavirus-covid-19-and-medical-devices/pooled-sample-testing-and-screening-testing-covid-19 and https://www.fda.gov/news-events/press-announcements/coronavirus-covid-19-update-fda-issues-first-emergency-authorization-sample-pooling-diagnostic accessed on 15 May 2021), sample pooling could be an important public health tool to increase testing capacity because it allows for more individuals to be analyzed rapidly at once, using fewer testing resources [13,14,15,16]. Moreover, this may be even more relevant if the screening involves a community, such as a school or a faculty returning to work or class, in order to promptly implement preventive measures if needed. In our study, we used a simple monodimensional pooling strategy to screen 1195 students and workers at the Università degli Studi del Sannio (University of Sannio, Benevento, Campania, Italy). After collection and heating-inactivation, aliquots of three samples were grouped in a batch before performing a multiplex RT-qPCR develop in-house targeting viral RdRP and N genes. Suspected positive triplets were then deconvoluted and analyzed individually. The effectiveness of the pooled testing strategy was next confirmed by using a commercially available IVD kit, demonstrating that test grouping could be a feasible and sustainable approach in environmental monitoring, especially when laboratories are overwhelmed by high demand for disease diagnosis.

## 2. Materials and Methods

### 2.1. Informed Consent

A screening campaign targeting students and staff of the Università degli Studi del Sannio (Benevento, Campania, Italy) was organized by the Department of Science and Technology at the end of September 2020 in collaboration with the Local Health Authority (Azienda Sanitaria Locale—ASL—Benevento, Italy) and the Municipality of Benevento (Prot. No. 15406—09/09/2020). Written informed consent was obtained from all participants. Collection of sensitive and personal data was done independently of molecular testing.

### 2.2. Swab Collection and Preparation

Naso-oropharyngeal swab specimens were collected and stored in sterile dry containers by trained staff in the cloister of Palazzo San Domenico, headquarters of the rectorate of the University. Swabs were then transported to the testing laboratory in refrigerated biocarriers. Under a BSL-2 laminar flow-cabinet (BioAir, Siziano-PV, Italy) respiratory specimens were treated with 200 μL of a solution of TE with 2.5 μL of MagMAX™ Viral/Pathogen Proteinase K (Applied Biosystems, Waltham, MA USA) by vigorously vortexing for 1 min followed by a heat inactivation step at 95 °C for 10 min. Then, samples were briefly spun down and batches were prepared by pooling 10 μL from three, five or seven different extracts. Next, 4 μL from the single extracts, or from batches of different sample combinations, were directly used as input in a multiplex RT-qPCR. Raw extracts from all swabs were stored at −80 °C until further analysis. All procedures (collection, transport, storage, and processing) were carried out in compliance with the appropriate safety precautions. Unpaired *t* tests with Welch’s correction were performed on GraphPad Prism 8.0.1 (GraphPad Software, San Diego, CA, USA) to determine statistical significance of differences in Ct values observed between negative samples or pools and positive sample or pools.

### 2.3. SARS-CoV-2 RT-qPCR

To pilot our strategy, we preliminarily developed a multiplex RT-qPCR protocol for the detection of the *RdRP* and *N* genes of SARS-CoV-2 in extracts from nasopharyngeal specimen collected retrospectively from 27 positive and 26 negative-diagnosed patients. Detection of SARS-CoV-2 in respiratory specimen was performed by multiplex RT-qPCR, through specific amplification of viral *N* and *RdRP* genes and the human *RNAseP (RP)* gene as an internal control. As positive controls, a synthetic DNA was used for *N*, whereas retrospective samples from positive patients were used for *RdRP* amplification. Sequences of CDC- and WHO-approved primers and dual-labeled probes for target genes were the following (Table 1).

Reverse transcription and amplification were carried out with the Reliance One-Step Multiplex RT-qPCR Supermix (Bio-Rad Laboratories, Hercules, CA, USA) in a 12 μL final volume reaction. RT-qPCR analyses were performed in both QuantStudio™ 5 System (Applied Biosystems, Waltham, MA USA) and CFX96 Touch Real Time PCR Detection System (Bio-Rad Laboratories, Hercules, CA, USA) with the following thermal cycling conditions: 50 °C for 10 min for reverse transcription, followed by 95 °C for 10 min and 45 cycles of 95 °C for 10 s and 61 °C for 30 s. Target amplification to detectable levels was measured by the QuantStudio™ 5 Design & Analysis Software v1.5.1 (Applied Biosystems, Waltham, MA USA) for each channel by comparing the ∆Rn of a sample well to a ∆Rn threshold. Baseline threshold was set at 100 RFU. A sample or a batch was considered positive or “suspected positive” for any Ct value of *RdRP* (Cy5) amplification within the 45 cycles or if the Ct value for *N1* (FAM/VIC) was lower than 32. Negative and positive samples were confirmed by using the IVD kit SARS-CoV-2 Real Time (Nuclear Laser Medicine s.r.l., Settala MI, Italy), which specifically targets viral *RdRP/Hel* [17], *N* and *E* (Envelope) genes. According to manufacturer’s instructions, a sample is positive for SARS-CoV-2 infection if at least one between *N* and *RdRP/Hel* is specifically amplified before the 40th cycle. The *E* target is used as a common feature of betacoronavirus. Each sample was tested at least once for each of the three test modes (pooled, through IHD- and IVD-assay). Graphs and statistics were done using GraphPad Prism 8.0.1.

## 3. Results

**Figure 1:**
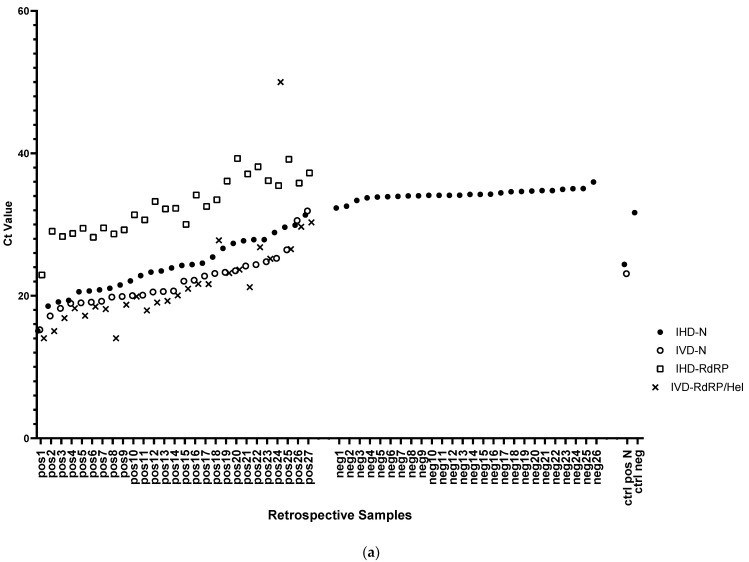

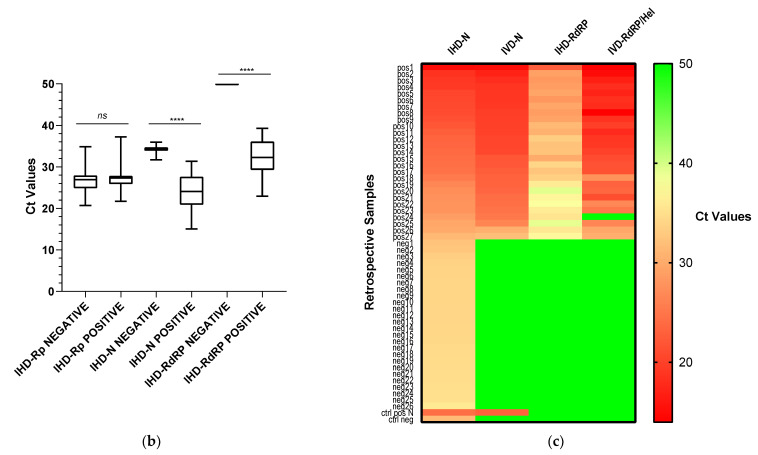
Pilot set up of SARS-CoV-2 detection by multiplex RT-qPCR on retrospective samples. (**a**) Scatter plot of Ct values for viral *N* and *RdRP* targets in 26 negative and 27 positive-retrospectively diagnosed patients detected by in-house developed (IHD) and IVD multiplex RT-qPCR. (**b**) Box and whiskers plot representing the differences of Ct values for *RP*, *N* and *RdRP* targets between positive and negative samples. Statistical significance of Ct value differences between negative and positive samples were measured with unpaired Welch’s *t* tests (****: *p* value < 0.0001; ns: not significant). (**c**) Heat map representing IHD-Ct values in comparison to amplification signals obtained at time of diagnosis through commercially available kit suitable for in vitro diagnostic use (IVD).

In a pilot stage, detection of SARS-CoV-2 was set up by analyzing 53 retrospective samples collected between August and September 2020 from 26 negative and 27 positively diagnosed patients with an in-house developed (IHD) multiplex assay targeting viral *N* and *RdRP* genes and human *RP* as internal amplification control. All retrospective samples showed Ct values for internal control *RP* ranging from 20.70 to 37.21. The positive samples showed amplification with Ct values less than 40 for the *RdRP* target and less than 32 for the *N* target. In contrast, negative samples showed no amplification for *RdRP*, and a nonspecific amplification in *N* [18], with Ct values ranging from 32.32 to 35.97 (Figure 1a). According to unpaired Welch’s *t* tests, differences in Ct values for *N* and *RdRP* between positive samples were statistically significant (*p* values ≤ 0.0001; Figure 1b).

Next, for each target we compared Ct values obtained from the IHD-multiplex assay with those recorded at the time of diagnosis with a commercially available IVD-validated kit. Among positives, the mean Ct value differences between IHD and IVD-multiplex assays were +2.05 ± 1.28 for *N* and +11.44 ± 2.61 for *RdRP*, although IVD-kit and IHD-kit target different regions of *RdRP* (Figure 1a,c).

**Figure 2:**
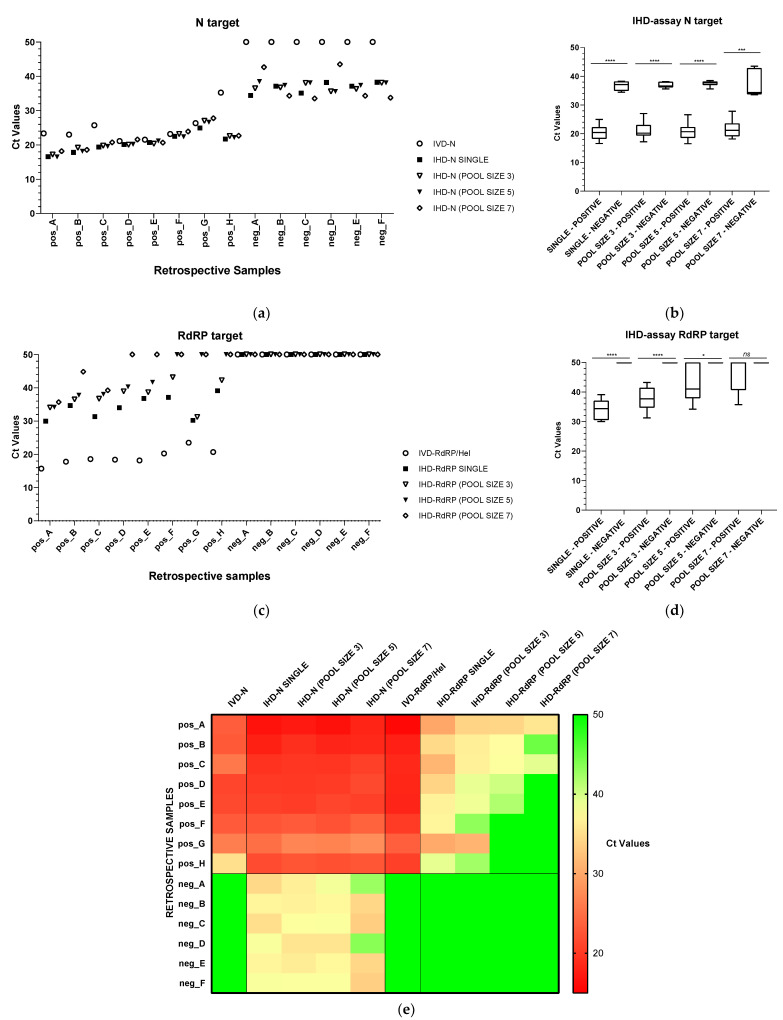
SARS- CoV-2 detection by multiplex RT-qPCR on pooled retrospective samples. Eight retrospective positive samples with high and low Ct values in *N* and in *RdRP* were grouped with six different negative samples in pool size of 3, 5 and 7. (**a**) Scatter plot representing Ct values recorded by using IVD or IHD assays targeting *N* gene on single samples or pooled samples. (**b**) Box and whiskers plot representing Ct differences for *N* target between positive and negative pools of different sizes. (**c**) Scatter plot representing Ct values recorded by using IVD or IHD assays targeting the *RdRP* gene on single samples or pooled samples. (**d**) Box and whiskers plot representing Ct dispersion differences for *RdRP* target between positive and negative pools of different sizes. (**e**) Heat map comparing Ct values for target genes *N* and *RdRp* recorded for eight positive and six negative retrospective samples through IVD-assay, or IHD-assay run as single sample or as pools of 3, 5 or 7. Statistical significance of Ct value differences between negative and positive samples or pools was measured with unpaired Welch’s t tests (****: *p* value < 0.0001; ***: *p* value = 0.0001; * *p* value < 0.02; ns: not significant).

In the pooling validation step, we selected eight positive samples with high and low Ct values in *N* and in *RdRP* ranging from 21.14 to 35.26, and from 15.70 to 23.52, respectively, and pooled them after extraction either with two different negative samples (pool size 3), or with four different negative samples (pool size 5), or with six different negative samples (pool size 7). In all pools with positive samples, we could detect *N* amplification, with Ct values ranging from 16.58 to 27.80, whereas negative pools showed nonspecific amplification with Ct values ranging from 33.53 to 43.51 (Figure 2a,e). As assessed by Welch’s *t* test, average values of Ct for *N* in positive pools of all sizes were always significantly lower than negative pools (*p* values ≤ 0.0001; Figure 2b).

*RdRP* amplification was detected in all positive pools made of three samples (with Ct values ranging from 31.24 to 43.21), whereas in pools of five or of seven different raw extracts, positive samples were recognized in five pools (Ct range 34.18–41.70) and three pools (Ct range 35.73–44.85), respectively (Figure 2c,e). Significant differences between average values of Ct for *RdRP* in positive and negative pools were observed only in pools of three and, at a lesser extent, of five (Figure 2d).

Based on these data and considering a loss of sensitivity in larger pools, we decided to use pools of three for mass screening and established three deconvolution criteria. A pool was ungrouped in at least one of the following cases: (1) specific amplification of *N* with Ct values lower than 32; (2) specific amplification of *RdRP* with any Ct value; (3) nonspecific amplification or specific amplification with Ct values greater than 40 for *RP*.

**Figure 3:**
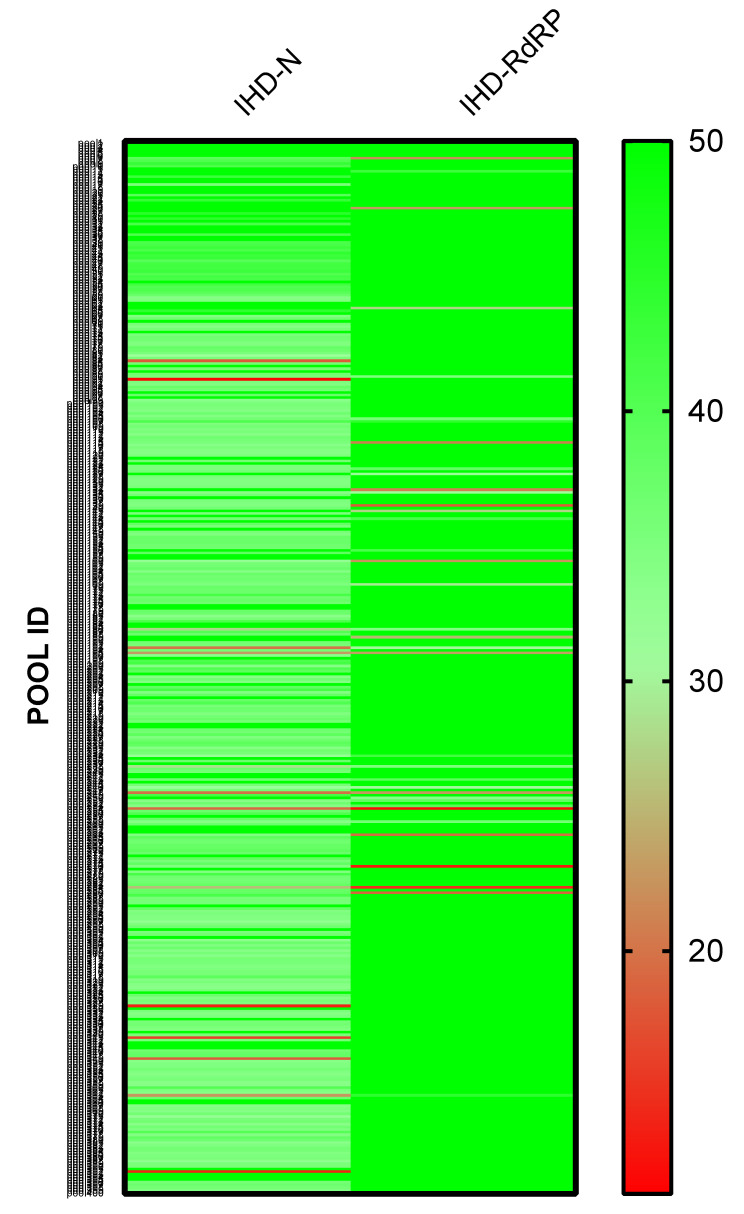
Heat map representing Ct values recorded by using an IHD multiplex assay for detection of SARS-CoV-2 *N* and *RdRP* genes on 400 pools obtained by grouping 1195 samples by three. Ct values of 50 were attribute to no amplified samples.

After obtaining written informed consent, respiratory swabs from 1195 asymptomatic individuals were collected and transported to the testing laboratory where the samples were soaked in TE buffer/Proteinase K, vortexed and boiled for 10 min at 95 °C, similar to other extraction-free protocols [19,20]. Uniquely labeled samples were used to form pools of three. Overall, 400 distinct pools were generated and analyzed in eight different runs. In each run, in addition to the appropriate controls, negative control triplets were included. All pools analyzed had Ct values for the internal control RP ranging from 12.22 to 30.29. Target amplification was observed in nine pools for *RdRP* with Ct values between 11.87 and 33.64, whereas seven pools (with no *RdRP* amplification) showed specific amplification of *N* with Ct values between 18.17 and 31.58 (Figure 3). In five additional pools, amplification for *RdRP* or *N* was inconclusive. Therefore, we deconvoluted 21 pools and analyzed singularly 63 samples with our IHD-multiplex assay. In the subsequent analysis step, only five samples had specific amplification for both *N* and *RdRP* with Ct values ranging from 19.36 to 33.76 and from 24.21 to 32.70, respectively, suggesting that our pooling strategy on raw extracts affected, somehow, amplification specificity.

**Figure 4:**
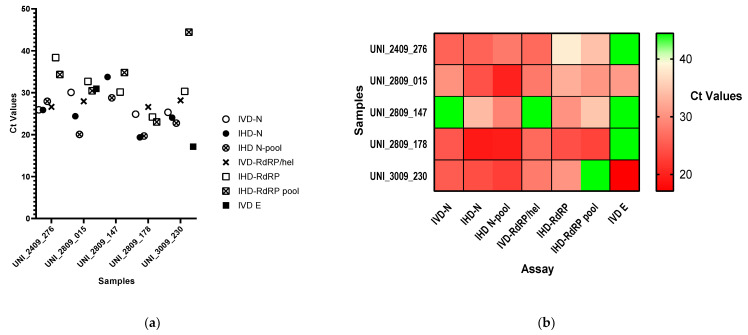
SARS-CoV-2 detection by multiplex RT-qPCR on deconvoluted positive samples. Scatter plot (**a**) and heat map (**b**) of Ct values recorded for each samples in distinct RT-qPCR assays.

The IVD-validated kit for detection of SARS-CoV-2 confirmed only four positive samples, whose Ct values are reported in Table 2 and represented in Figure 4. The sample UNI_2809_147 scored positive only for *RdRP* amplification through the IHD-assay (both singularly and in the pool) but was not confirmed by the IVD-multiplex assay. Interestingly, the differences in the Ct values observed when comparing the performance of the IHD, IVD, and pool assay for each sample or batch varied substantially, indicating that the quality of raw extracts may have affected the amplification outcome of each target differently. Moreover, lower Ct values observed in some positive pools than in single samples (in UNI_2409_276 for *RdRP*; in UNI_2809_015 for both *N* and *RdRP*; in UNI_2809_178 for *RdRP*; in UNI_3009_230 for *N*) were also recorded, probably due to the carrier effect of the high RNA content in the pooled samples [21].

To assess the accuracy of our sample pooling approach, we also analyzed singularly the samples from the negative triplets through IHD-multiplex assay and all resulted negative for SARS-CoV-2 genes and positive for internal control *RP* (data not shown). By comparison, during the revision of the manuscript, we have further analyzed single samples through IVD-multiplex assay, confirming previous negative diagnosis. Although the molecular analysis with the two assays was performed at two different periods on extracts stored at −80 °C, all samples showed no amplification for viral genes and specific amplification of internal controls.

In conclusion, considering that the mean prevalence of SARS-CoV-2 in Italy in October 2020 was about 0.09%, the accuracy of the IHD-assay evaluated on all deconvoluted samples was 99.92% (CI95 99.53–100.00%), with a sensitivity of 100.00% (CI95 39.76–100.00%) and a specificity of 99.92% (CI95 99.531–100.00%). The agreement between the IVD and the IHD-assays was described by a Cohen’s *k* value of 0.888 (CI95 0.671–1.000).

Within 24 h after swab collection, positive individuals were promptly contacted and reported to the relevant health authority. It is noteworthy that only one of them declared to have dysgeusia and anosmia, which are typical COVID19-related symptoms, whereas others were completely asymptomatic at the moment of swab collection.

In conclusion, thanks to our screening based on a simple monodimensional sample pooling strategy we were able to identify positive cases for SARS-CoV-2 among 1195 asymptomatic individuals using a sensitive gold standard approach for viral detection and, at the same time, substantially reducing time, costs and resources for diagnosis.

## 4. Discussion

Screening of large asymptomatic groups of individuals for SARS-CoV-2 infection through gold standard approaches, such as RNA extraction and RT-qPCR, can be expensive and wasteful when the prevalence of a given pathogen in the population is very low. Grouping strategies could be an important public health tool to increase testing capacity, reduce workload and contain reagent costs [6,7,13,15]. Sample pooling is already used as a successful cost-effective approach in screening for low-prevalence pathogens such as avian influenza (AI) H5N1 and human immunodeficiency viruses (HIV) [22,23].

In our work, we used a simple monodimensional pooling strategy for the detection of SARS-CoV-2 actively infected individuals among students and staff of the University of Sannio a few days before the beginning of academic lessons. With some approximations, we set up a screening method based on an extraction-free step followed by pooling of three samples at a time and analysis through an in house-developed (IHD) dual-labeled probe-based-multiplex RT-qPCR. In the pilot phase, we were able to first determine the ability of our assay to discriminate positive and negative retrospective samples and, secondly, its performance on pooled samples. We evaluated the performance of our IHD-assay on pools of different size. Although there was concordance on diagnosing retrospective samples singularly, or in three-sized pools based on both N and RdRP detection, pooling by five or seven resulted in a reduction in sensitivity for RdRP amplification.

Next, we grouped 1195 distinct samples from asymptomatic individuals in 400 pools and tested them by RT-qPCR. Consistent with preliminary observations, and according to three broad deconvolution criteria, we ungrouped 16 suspected pools plus five pools whose amplification was inconclusive and analyzed individually 63 samples with our IHD-multiplex assay. Out of 63, only five samples resulted positive, four of which were confirmed by an IVD-validated kit. This definitely low number of SARS-CoV-2-positive individuals identified in the community of the University of Sannio at the beginning of the academic year is consistent with previous seroprevalence studies indicating that early restrictions were successful in limiting COVID-19 diffusion in the district of Benevento [24,25].

The variations in Ct values for each target observed in different assays varied consistently, possibly due to several factors including the quality of the raw extracts, the small volumes used for batching, and, in the case of *RdRP*, by the fact that the IVD kit uses *RdRP/Hel* probe instead of *RdRP-P2* [8,17]. We could also observe earlier amplification of both *N* and *RdRP* in some positive pools with respect to single samples, probably due to the impact of the higher RNA content in the triplets. Large amounts of RNA in pools are also likely to increase nonspecific amplification, explaining the fair number of triplets requiring deconvolution [21].

A major limitation of this study is that in the pilot stage we did not evaluate at which extent raw extraction affects amplification results for each target in comparison to the classical purification procedures. Indeed, due to a lack of positive control plasmids, and due to a raw extraction with Proteinase K and heat, we were not able to evaluate the changes in sensitivity occurred in the pooling step in terms of viral copy number. The high impact of false-positive results after pool analysis was unexpected, suggesting that pooling conditions and deconvolution criteria could be improved by automating the extraction process and/or performing a multiplex fluorescence melting curve analysis [26] following RT-qPCR at the time of the screening, as Ct values could be biased by either poor-quality extraction or nonspecific amplification.

However, in comparison to a reference IVD-assay, our IHD-multiplex assay showed an accuracy of 99.92% (CI95 99.53–100.00%), with high sensitivity (100.00%; CI95 39.76–100.00%) and specificity (99.92%; CI95 99.531–100.00%) on all deconvoluted samples. Although we cannot formally exclude the possibility of a loss of power in detecting viral genes, due to the delay in confirming previous negative diagnosis through a IVD-multiplex assay, this possibility appears to be unlikely since internal controls were efficiently amplified in all samples.

Additionally, we conducted 463 analyses instead of 1195, reducing testing resources by more than 60% without lengthening diagnosis time and without significant losses in sensitivity. Although this saving can be further optimized by refining the pooling strategy set-up and improving analysis methods, this approach can contribute significantly to the rational use of human and material resources, especially in the context of public screening or for the implementation of environmental surveillance measures repeated over time in educational settings [6,27,28].

## Figures and Tables

**Table 1:** Primer and dual-labeled probe sequences used for multiplex RT-qPCR assays.

Primer/Probe Name	Sequence (5′→3′)
*2019-nCoV_N1-P*	[FAM/VIC]ACCCCGCATTACGTTTGGTGGACC[BHQ1]
*2019-nCoV_N1-F*	GACCCCAAAATCAGCGAAA
*2019-nCoV_N1-R*	TCTGGTTACTGCCAGTTGAATCTG
*hRP-Probe*	[HEX]TTCTGACCTGAAGGCTCTGCGCG[BHQ1]
*hRP-F*	AGATTTGGACCTGCGAGCG
*hRP-R*	GAGCGGCTGTCTCCACAAGT
*RdRP_SARSr-P2*	[CY5]CAGGTGGAACCTCATCAGGAGATGC[BBQ650]
*RdRP_SARSr-F2*	GTGARATGGTCATGTGTGGCGG
*RdRP_SARSr-R1*	CARATGTTAAASACACTATTAGCATA

**Table 2:** Comparison of Ct Values for SARS-CoV-2 targets in samples analyzed with IVD, IHD and IHD-pooled multiplex RT-qPCR assays.

Samples	CT Values							Positivity to SARS-CoV-2
	*N* IVD	*N* IHD	*N* IHD POOL	*RdRP/HEL* IVD	*RdRP* IHD	*RdRP* IHD POOL	*E* IVD	
UNI_2409_276	25.93	25.87	27.96	26.61	38.37	34.35	N/A	Confirmed
UNI_2809_015	30.06	24.39	20.06	27.95	32.70	30.45	30.94	Confirmed
UNI_2809_147	N/A	33.76	28.77	N/A	30.15	34.82	N/A	Not confirmed
UNI_2809_178	24.89	19.36	19.67	26.59	24.21	23.02	N/A	Confirmed
UNI_3009_230	25.33	24.05	22.74	28.17	30.33	44.44	17.13	Confirmed

## Data Availability

Data are available upon request from corresponding authors.

## Abbreviations

SARS-CoV-2	Severe Acute Respiratory Syndrome Coronavirus 2
WHO	World Health Organization
RT-qPCR	Reverse Transcription-Quantitative Polymerase Chain Reaction
IVD	For In Vitro Diagnostic use
IHD	In-House developed
COVID-19	Coronavirus Disease of 2019
*RP*	*RNAseP* gene
*RdRP*	*RNA-dependent RNA Polymerase* gene
*RdRP/Hel*	*RNA-dependent RNA Polymerase/Helicase* gene
*N*	*Nucleocapsid Protein* gene
*E*	*Envelope* gene
BSL-2	Biosafety Level 2
CDC	Center for Disease Control and Prevention
